# Supplementary material for: Methane stimulates massive nitrogen loss from freshwater reservoirs in India
Source: Nat Commun. 2018 Mar 28;9:1265. doi: 10.1038/s41467-018-03607-z (PMC5871758; doi:10.1038/s41467-018-03607-z)
Supplement: Supplementary file 1 — Supplementary Information(PDF 1872 kb) [file 41467_2018_3607_MOESM1_ESM.pdf]

Supplementary Information

Methane stimulates massive nitrogen loss from freshwater reservoirs in India

Naqvi, *et. al.*

Correspondence to: wajih_naqvi@yahoo.com

This PDF file contains:

Supplementary Figures 1 to 14

Supplementary Tables 1 to 5

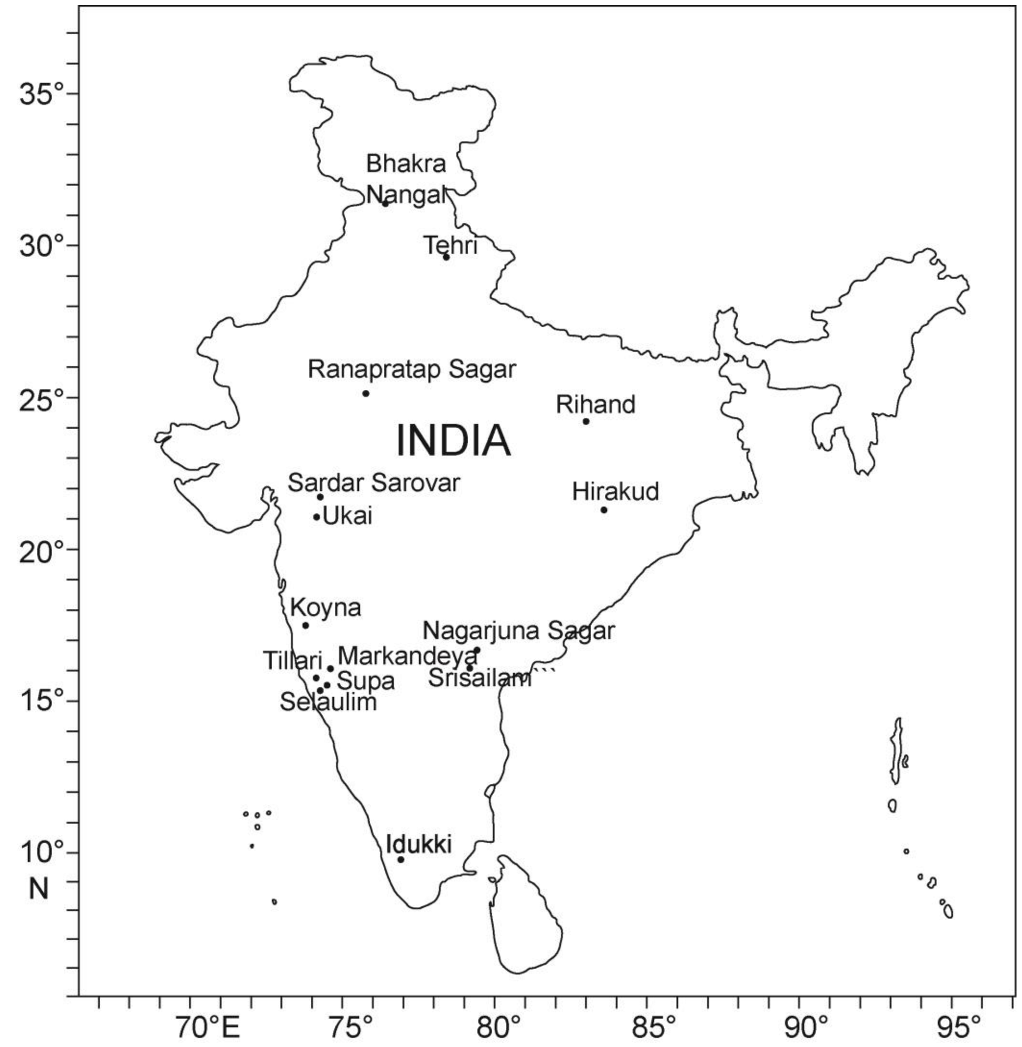

Supplementary Figure 1. Locations of dam reservoirs

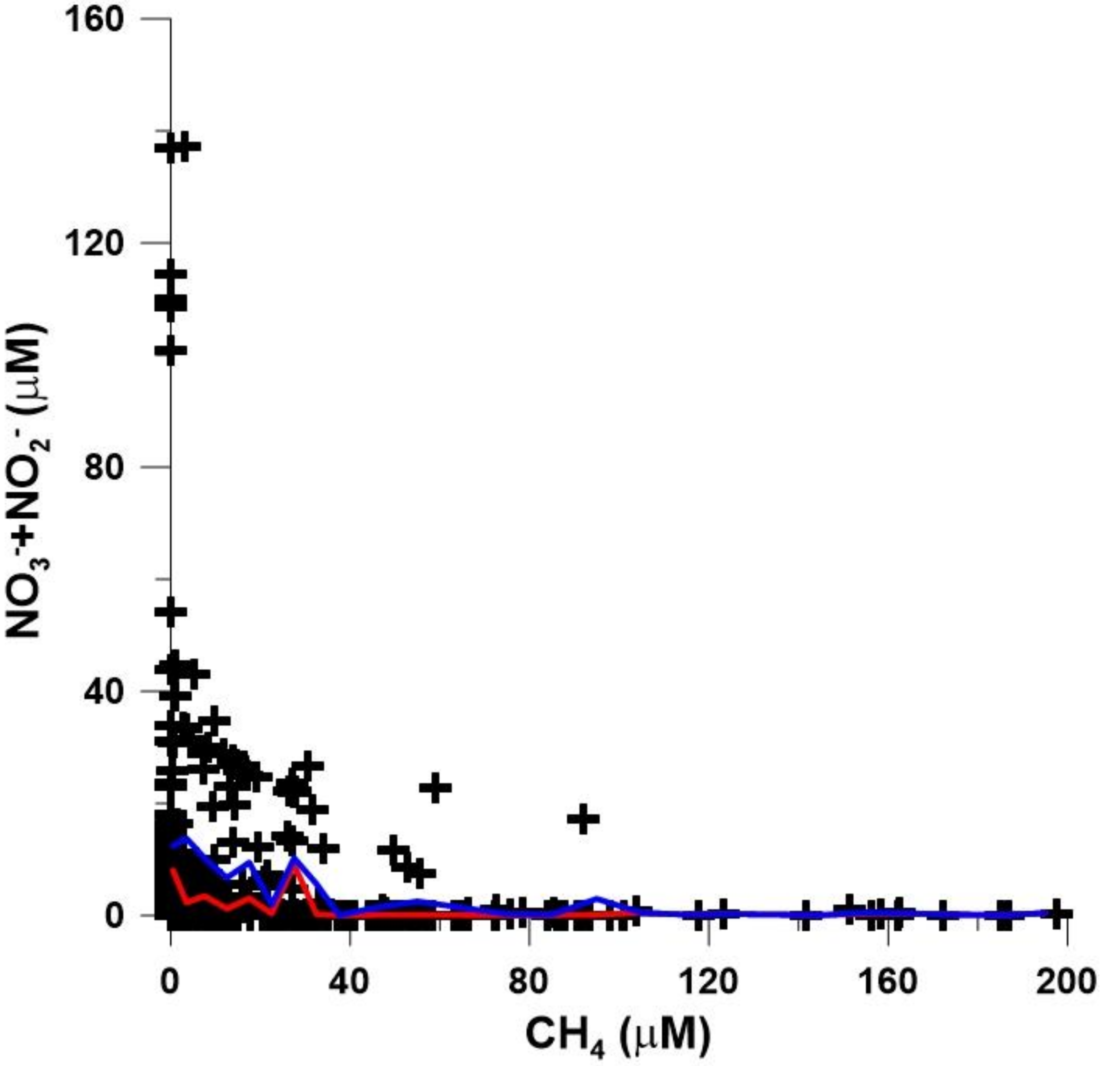

Supplementary Figure 2. Plot of nitrate+nitrite versus methane in oxygen-depleted (O_2<2 ml l^{-1}) hypolimnia of all reservoirs (n=325). The blue and red lines connect median and mean nitrate+nitrite concentrations for the following methane concentration ranges: <0.5 μM; 0.5-5 μM; 5-40 μM with 5 μM interval; 40-200 μM with 10 μM interval.

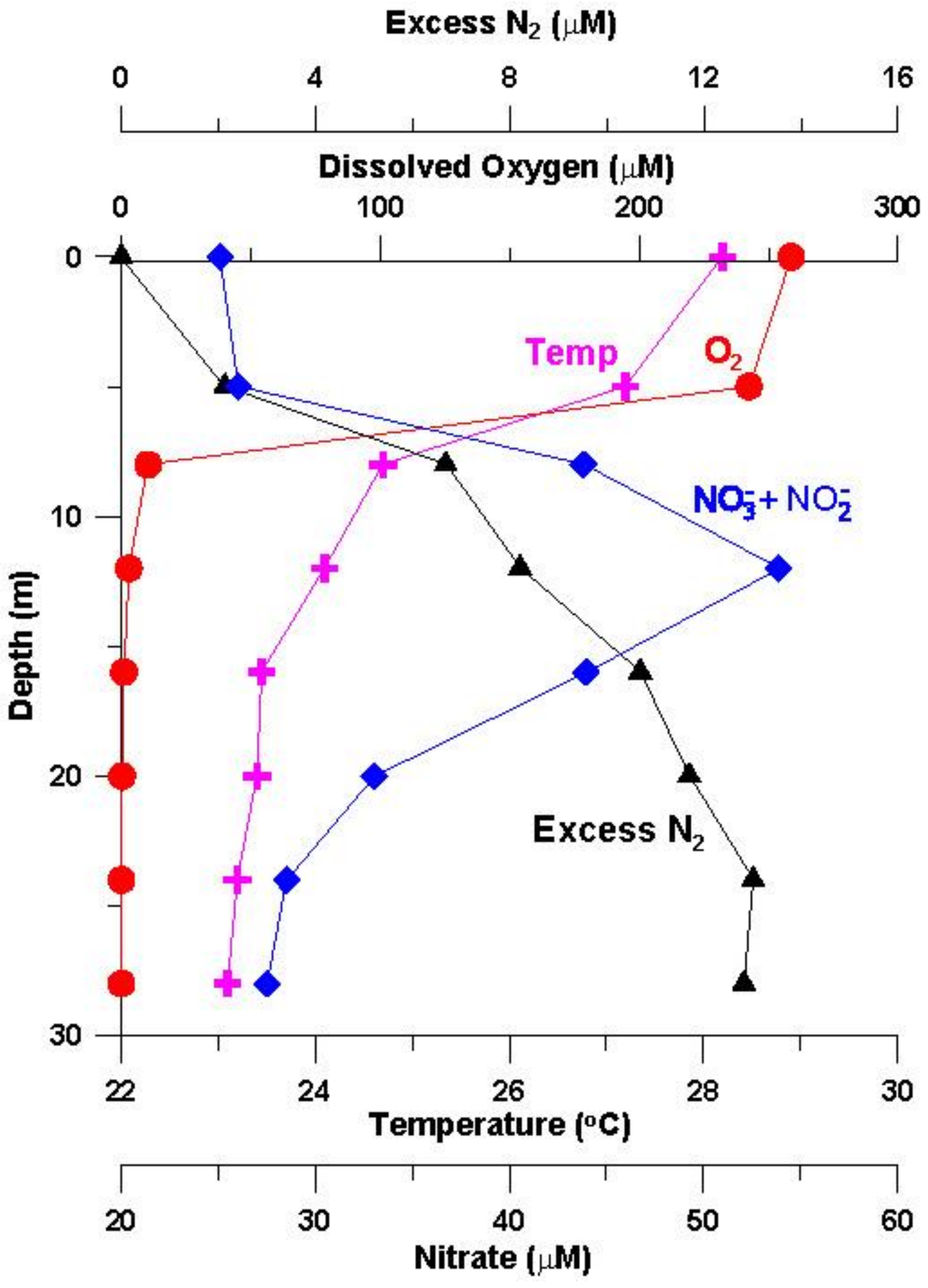

Supplementary Figure 3. Vertical profiles of temperature, dissolved oxygen, nitrate+nitrite, and excess N_2 computed from the N_2/Ar ratio in Markandeya Reservoir on 17.04.2015.

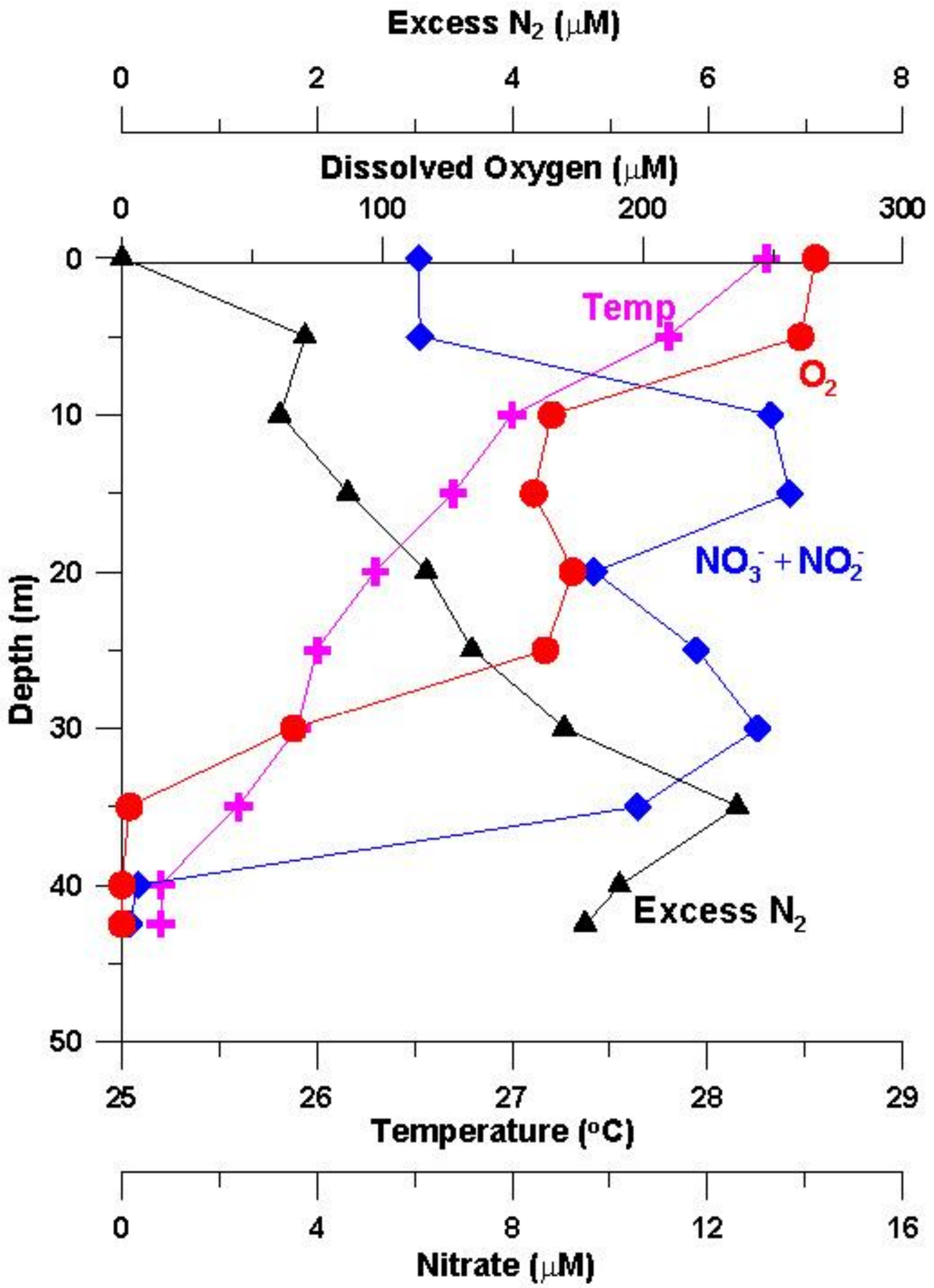

Supplementary Figure 4. Vertical profiles of temperature, dissolved oxygen, nitrate+nitrite, and excess N_2 computed from the N_2/Ar ratio in Tillari Reservoir on 21.07.2015.

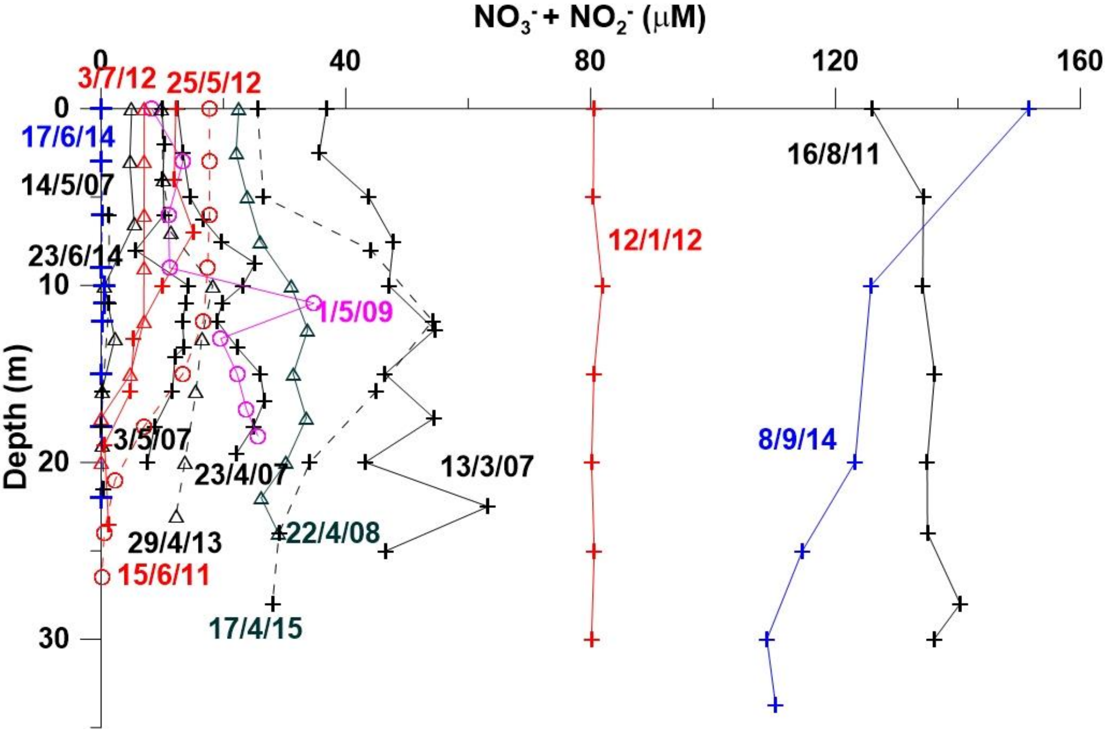

Supplementary Figure 5. Depth profiles of nitrate (including traces of nitrite) on 16 trips to Markandeya Reservoir.

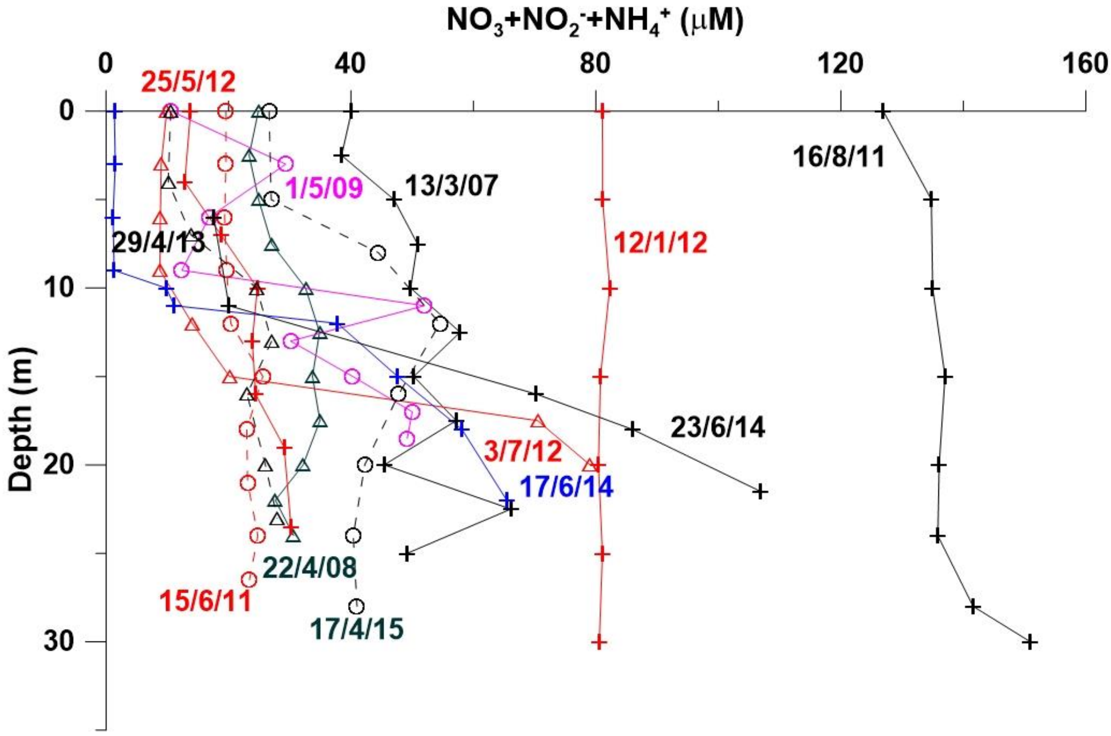

Supplementary Figure 6. Depth profiles of dissolved inorganic nitrogen (nitrate+nitrite+ammonium) on 12 trips to Markandeya Reservoir. Ammonium was not measured on 4 trips because of technical difficulties.

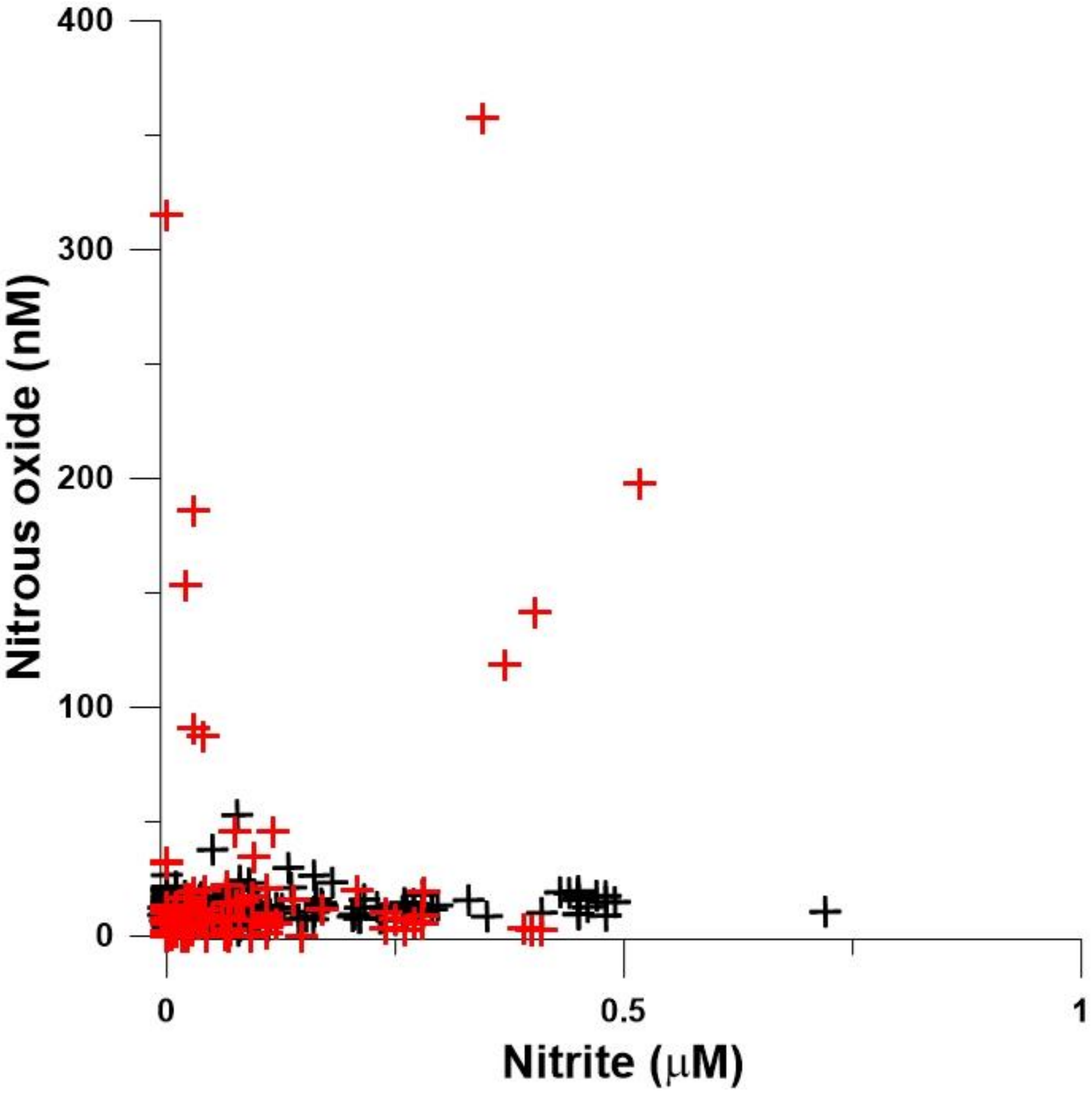

Supplementary Figure 7. Plot of nitrous oxide versus nitrite in Tillari Reservoir. Red (black) symbols denote samples having oxygen contents of less (more) than 0.5 ml l^{-1}.

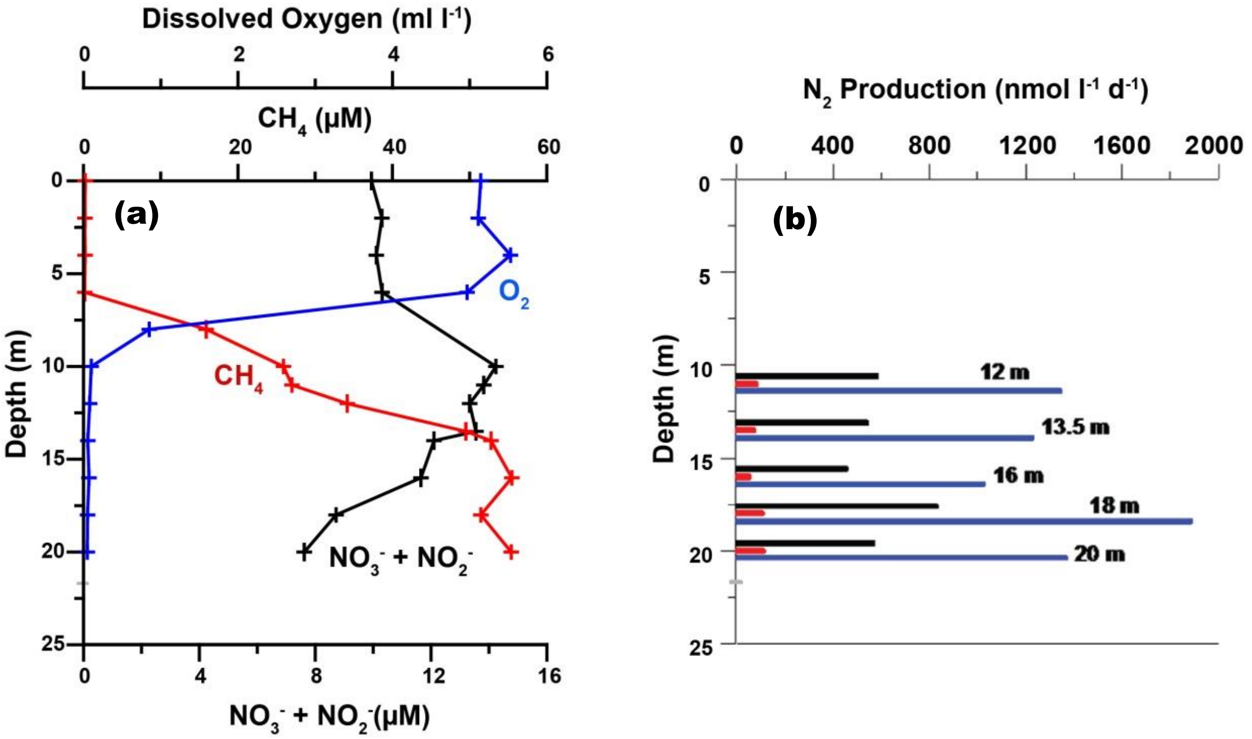

Supplementary Figure 8. Depthwise variations in concentrations of dissolved oxygen, nitrate+nitrite and methane (a), and N_2 production rates measured by isotope pairing experiments (b): black bars - $^{15}N^{15}N$; red bars - $^{15}N^{14}N$; blue bars - total denitrification rate) in Markandeya Reservoir on 03.05.2007.

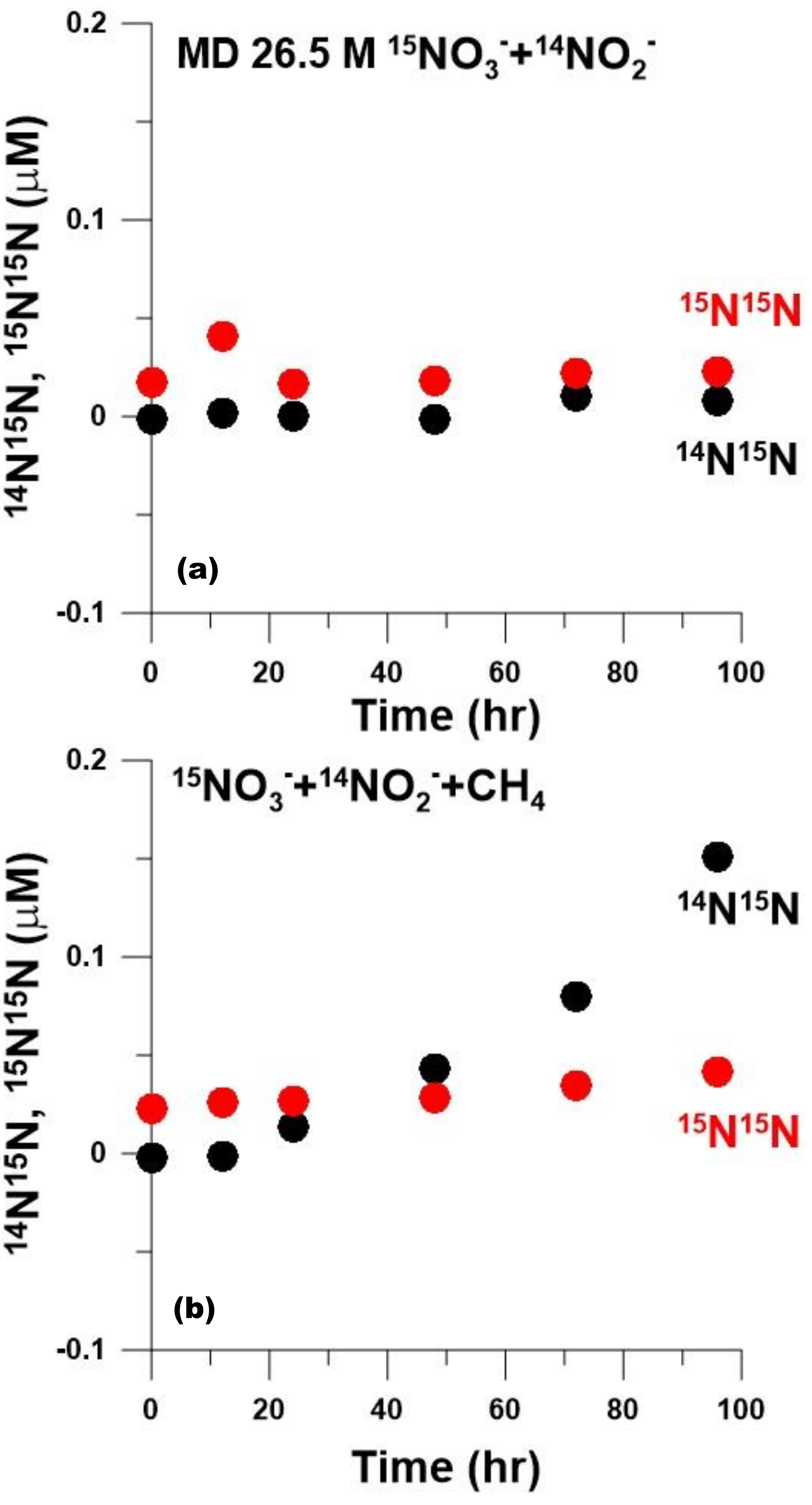

Supplementary Figure 9. Changes in concentrations of $^{14}N^{15}N$ and $^{15}N^{15}N$ versus time in a water sample collected from 26.5 m depth in Markandeya Reservoir on 15.06.2011 and incubated with $^{15}NO_3^-$+$^{14}NO_2^-$ in the absence (a) and presence (b) of methane.

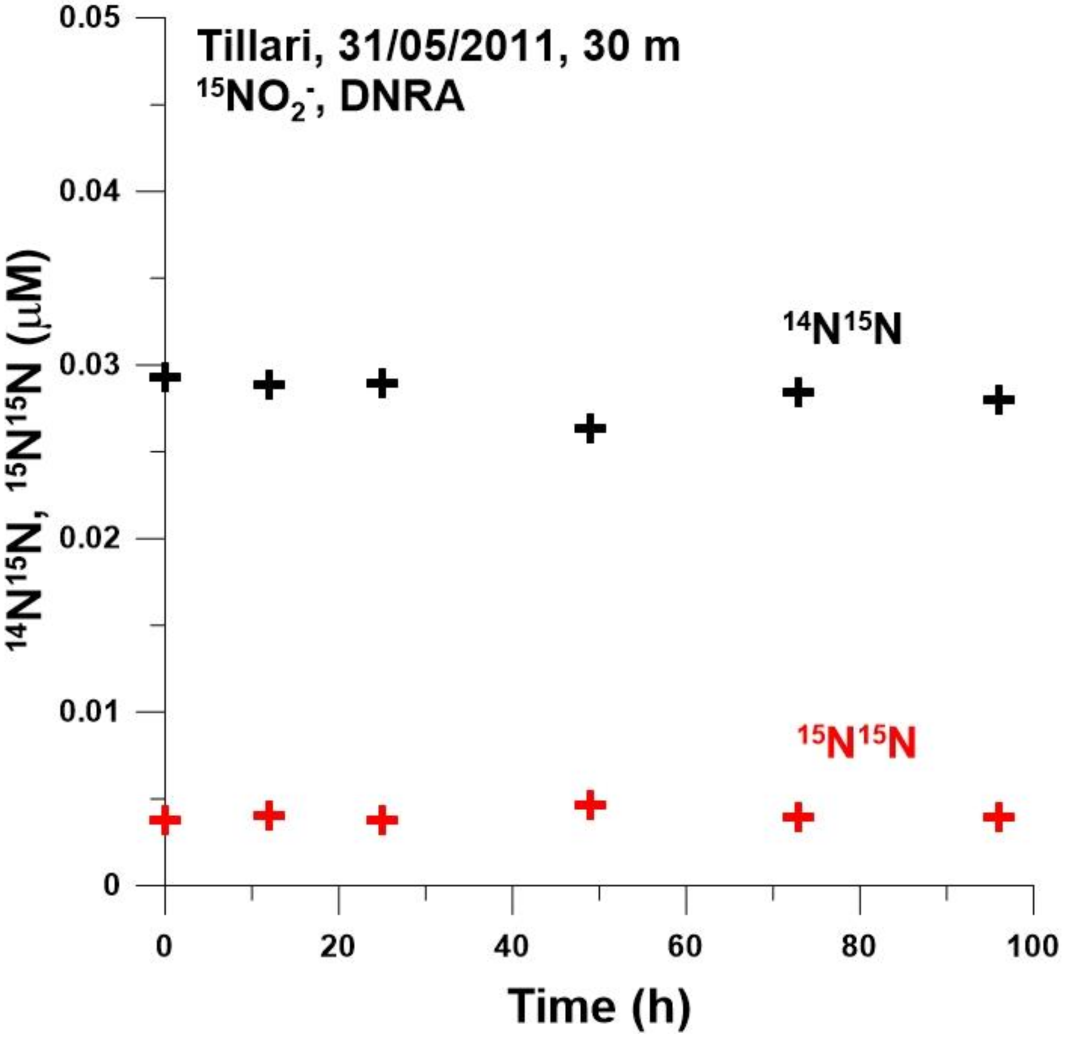

Supplementary Figure 10. Lack of DNRA activity as demonstrated by the absence of significant labelled NH_4^+ production in a water sample collected from 30 m depth in Tillari Reservoir on 31.05.2011 and incubated with $^{15}NO_2^-$. NH_4^+ present in solution was subsequently converted into N_2 by adding NaOBr.

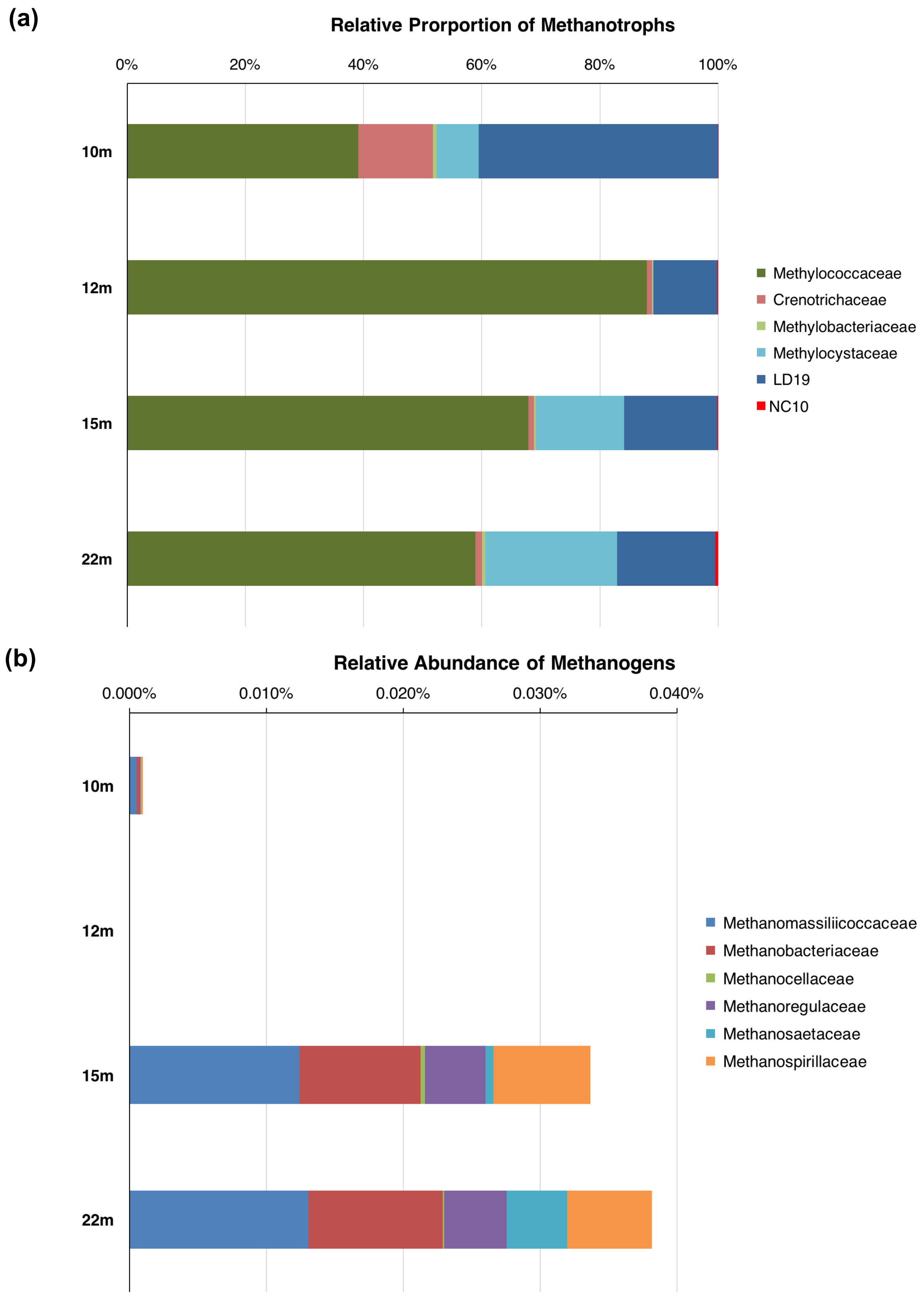

Supplementary Figure 11. (a) Relative proportion of known methanotrophs, and (b) relative abundance of methanogens detected in amplicon sequencing of 16S rRNA genes in Markandeya Reservoir on 17.06.2014. All shown are at the family level except for the phylum NC10.

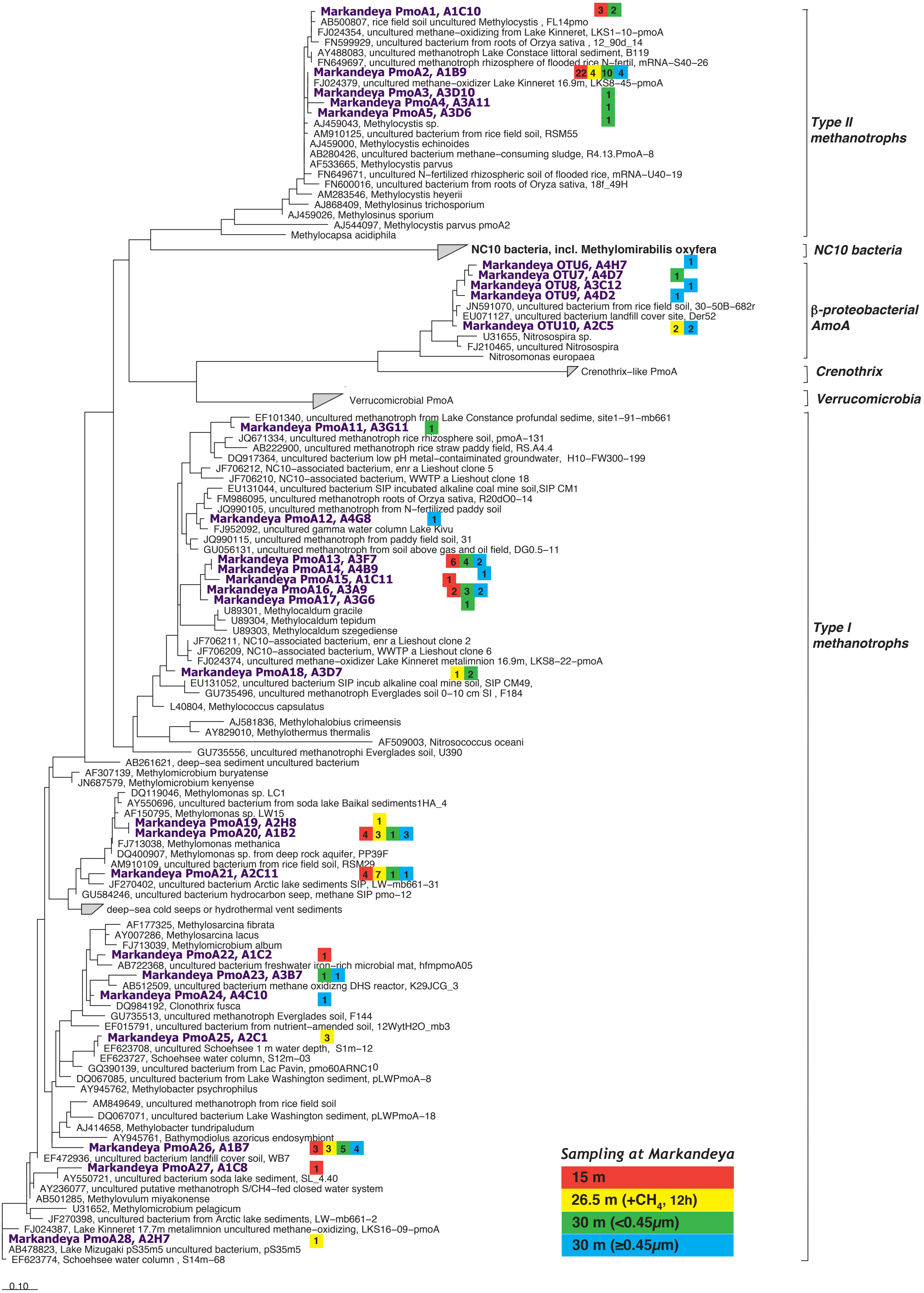

Markandeya PmoA1, A1C10
3 2
AB500807, rice field soil uncultured Methylocystis , FL14pmo
FJ024354, uncultured methane−oxidizing from Lake Kinneret, LKS1−10−pmoA
FN599929, uncultured bacterium from roots of Orzya sativa , 12_90d_14
AY488083, uncultured methanotroph Lake Constace littoral sediment, B119
FN649697, uncultured methanotroph rhizosphere of flooded rice N−fertil, mRNA−S40−26
Markandeya PmoA2, A1B9
22 4 10 4
FJ024379, uncultured methane−oxidizer Lake Kinneret 16.9m, LKS8−45−pmoA
Markandeya PmoA3, A3D10
1
Markandeya PmoA4, A3A11
1
Markandeya PmoA5, A3D6
1
AJ459043, Methylocystis sp.
AM910125, uncultured bacterium from rice field soil, RSM55
AJ459000, Methylocystis echinoides
AB280426, uncultured bacterium methane−consuming sludge, R4.13.PmoA−8
AF533665, Methylocystis parvus
FN649671, uncultured N−fertilized rhizospheric soil of flooded rice, mRNA−U40−19
FN600016, uncultured bacterium from roots of Oryza sativa, 18f_49H
AM283546, Methylocystis heyerii
AJ868409, Methylosinus trichosporium
AJ459026, Methylosinus sporium
AJ544097, Methylocystis parvus pmoA2
Methylocapsa acidiphila
Type II methanotrophs
NC10 bacteria, incl. Methylomirabilis oxyfera
NC10 bacteria
Markandeya OTU6, A4H7
1
Markandeya OTU7, A4D7
1
Markandeya OTU8, A3C12
1
Markandeya OTU9, A4D2
1
JN591070, uncultured bacterium from rice field soil, 30−50B−682r
EU071127, uncultured bacterium landfill cover site, Der52
Markandeya OTU10, A2C5
2 2
U31655, Nitrosospira sp.
FJ210465, uncultured Nitrosospira
Nitrosomonas europaea
β-proteobacterial AmoA
Crenothrix−like PmoA
Crenothrix
Verrucomicrobial PmoA
Verrucomicrobia
EF101340, uncultured methanotroph from Lake Constance profundal sedime, site1−91−mb661
Markandeya PmoA11, A3G11
1
JQ671334, uncultured methanotroph rice rhizosphere soil, pmoA−131
AB222900, uncultured methanotroph rice straw paddy field, RS.A4.4
DQ917364, uncultured bacterium low pH metal−contaiminated groundwater, H10−FW300−199
JF706212, NC10−associated bacterium, enr a Lieshout clone 5
JF706210, NC10−associated bacterium, WWTP a Lieshout clone 18
EU131044, uncultured bacterium SIP incubated alkaline coal mine soil,SIP CM1
FM986095, uncultured methanotroph roots of Orzya sativa, R20dO0−14
JQ990105, uncultured methanotroph from N−fertilized paddy soil
Markandeya PmoA12, A4G8
1
FJ952092, uncultured gamma water column Lake Kivu
JQ990115, uncultured methanotroph from paddy field soil, 31
GU056131, uncultured methanotroph from soil above gas and oil field, DG0.5−11
Markandeya PmoA13, A3F7
6 4 2
Markandeya PmoA14, A4B9
1
Markandeya PmoA15, A1C11
1
Markandeya PmoA16, A3A9
2 3 2
Markandeya PmoA17, A3G6
1
U89301, Methylocaldum gracile
U89304, Methylocaldum tepidum
U89303, Methylocaldum szegediense
JF706211, NC10−associated bacterium, enr a Lieshout clone 2
JF706209, NC10−associated bacterium, WWTP a Lieshout clone 6
FJ024374, uncultured methane−oxidizer Lake Kinneret metalimnion 16.9m, LKS8−22−pmoA
Markandeya PmoA18, A3D7
1 2
EU131052, uncultured bacterium SIP incub alkaline coal mine soil, SIP CM49,
GU735496, uncultured methanotroph Everglades soil 0−10 cm SI , F184
L40804, Methylococcus capsulatus
AJ581836, Methylohalobius crimeensis
AY829010, Methylothermus thermalis
AF509003, Nitrosococcus oceani
GU735556, uncultured methanotrophi Everglades soil, U390
AB261621, deep−sea sediment uncultured bacterium
AF307139, Methylomicrobium buryatense
JN687579, Methylomicrobium kenyense
DQ119046, Methylomonas sp. LC1
AY550696, uncultured bacterium from soda lake Baikal sediments1HA_4
AF150795, Methylomonas sp. LW15
Markandeya PmoA19, A2H8
1
Markandeya PmoA20, A1B2
4 3 1 3
FJ713038, Methylomonas methanica
DQ400907, Methylomonas sp. from deep rock aquifer, PP39F
AM910109, uncultured bacterium from rice field soil, RSM29
Markandeya PmoA21, A2C11
4 7 1 1
JF270402, uncultured bacterium Arctic lake sediments SIP, LW−mb661−31
GU584246, uncultured bacterium hydrocarbon seep, methane SIP pmo−12
deep−sea cold seeps or hydrothermal vent sediments
AF177325, Methylosarcina fibrata
AY007286, Methylosarcina lacus
FJ713039, Methylomicrobium album
Markandeya PmoA22, A1C2
1
AB722368, uncultured bacterium freshwater iron−rich microbial mat, hfmpmoA05
Markandeya PmoA23, A3B7
1 1
AB512509, uncultured bacterium methane oxidizng DHS reactor, K29JCG_3
Markandeya PmoA24, A4C10
1
DQ984192, Clonothrix fusca
GU735513, uncultured methanotroph Everglades soil, F144
EF015791, uncultured bacterium from nutrient−amended soil, 12WytH2O_mb3
Markandeya PmoA25, A2C1
3
EF623708, uncultured Schoehsee 1 m water depth, S1m−12
EF623727, Schoehsee water column, S12m−03
GQ390139, uncultured bacterium from Lac Pavin, pmo60ARNC10
DQ067085, uncultured bacterium from Lake Washington sediment, pLWPmoA−8
AY945762, Methylobacter psychrophilus
AM849649, uncultured methanotroph from rice field soil
DQ067071, uncultured bacterium Lake Washington sediment, pLWPmoA−18
AJ414658, Methylobacter tundripaludum
AY945761, Bathymodiolus azoricus endosymbiont
Markandeya PmoA26, A1B7
3 3 5 4
EF472936, uncultured bacterium landfill cover soil, WB7
Markandeya PmoA27, A1C8
1
AY550721, uncultured bacterium soda lake sediment, SL_4.40
AY236077, uncultured putative methanotroph S/CH4−fed closed water system
AB501285, Methylovulum miyakonense
U31652, Methylomicrobium pelagicum
JF270398, uncultured bacterium from Arctic lake sediments, LW−mb661−2
FJ024387, Lake Kinneret 17.7m metalimnion uncultured methane−oxidizing, LKS16−09−pmoA
Markandeya PmoA28, A2H7
1
AB478823, Lake Mizugaki pS35m5 uncultured bacterium, pS35m5
EF623774, Schoehsee water column , S14m−68
Type I methanotrophs
Sampling at Markandeya
15 m
26.5 m (+CH4, 12h)
30 m (<0.45μm)
30 m (≥0.45μm)
0.10

Supplementary Figure 12. A phylogenetic reconstruction of particulate monooxygenase gene based on deduced amino acid sequences obtained in the Markandeya Reservoir (purple) in this study. Shown here is the best maximum likelihood (ML) tree after 100 resamplings and its topology is supported by maximum parsimony and distance matrix methods. Only sequences of ≥ amino acids long were used in tree construction, and only representative OTU sequences (97% nuceic acids sequence similarity cutoff) are shown here. Coloured rectangles denote the sampling depths at which sequences were obtained, and the numbers within indicate the number of sequences yielded of the OTU at the corresponding sampling depths. Branch lengths are in sequence divergence.

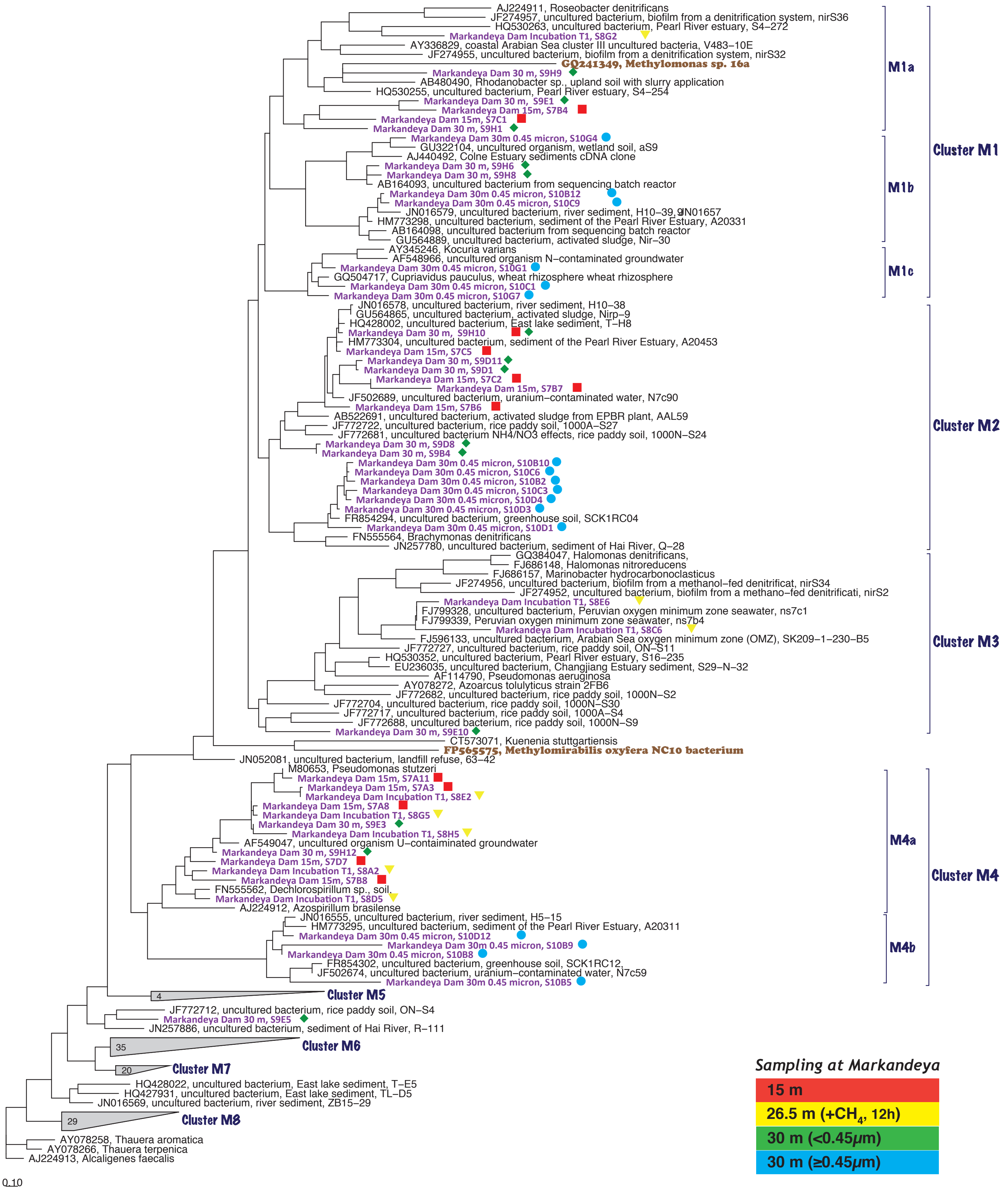

Supplementary Figure 13. A phylogenetic recontruction of cd1-containing nitrite reductase (NirS) based on deduced amino acid sequences obtained in the Markandeya Reservoir (purple). Shown here is the best maximum likelihood (ML) tree after 100 resamplings and its topology is supported by both maximum parsimony and distance matrix methods. Only sequences of ≥220 amino acids long were used in tree construction, and only representative OTU sequences (97% nucleic acids sequence similarity cutoff) are shown here. Coloured rectangles denote the sampling depths at which sequences were obtained, and the numbers within indicate the number of sequences yielded of that OTU at the corresonding sampling depths. Branch lengths are in sequence divergence.

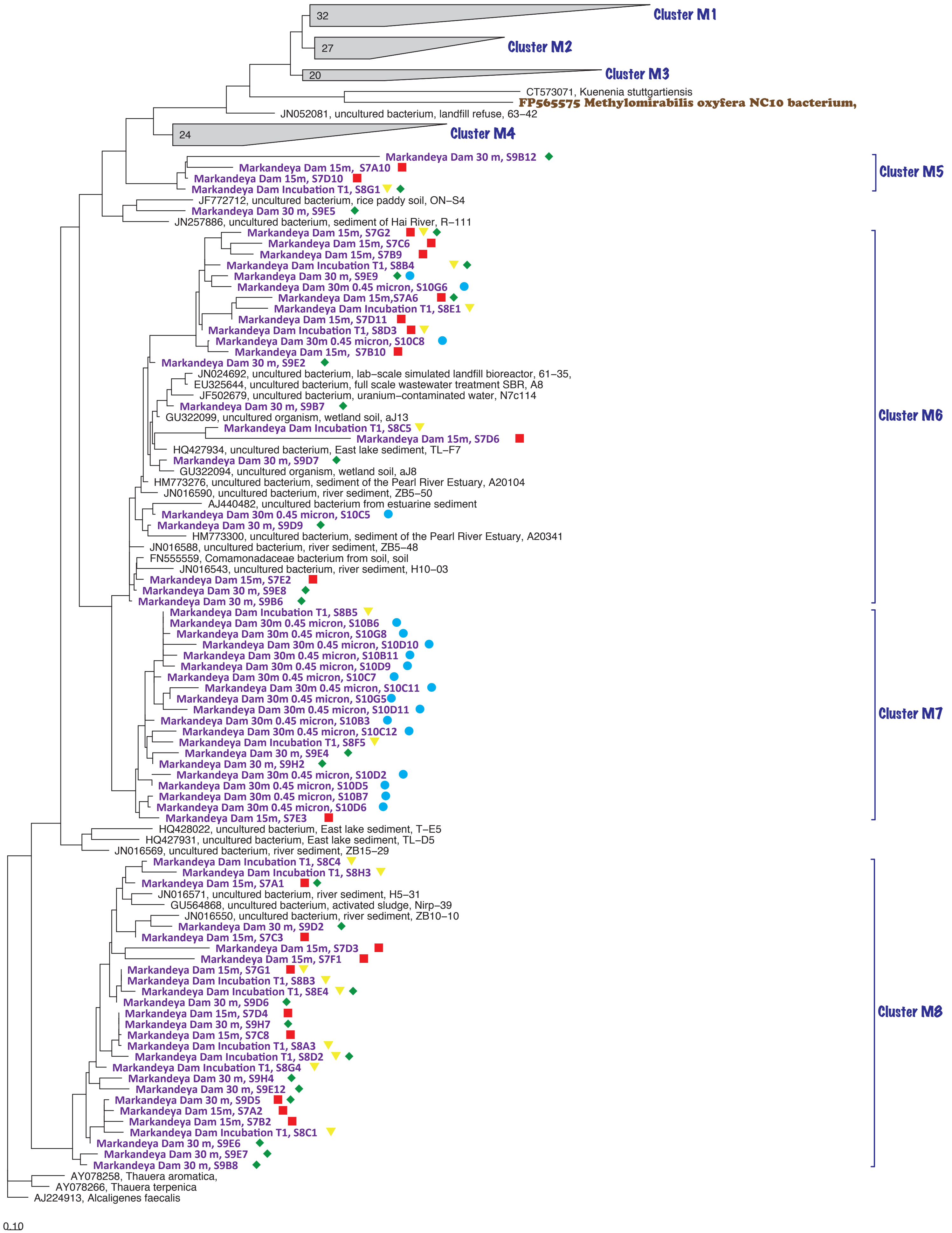

Supplementary Figure 13 (cont'd)

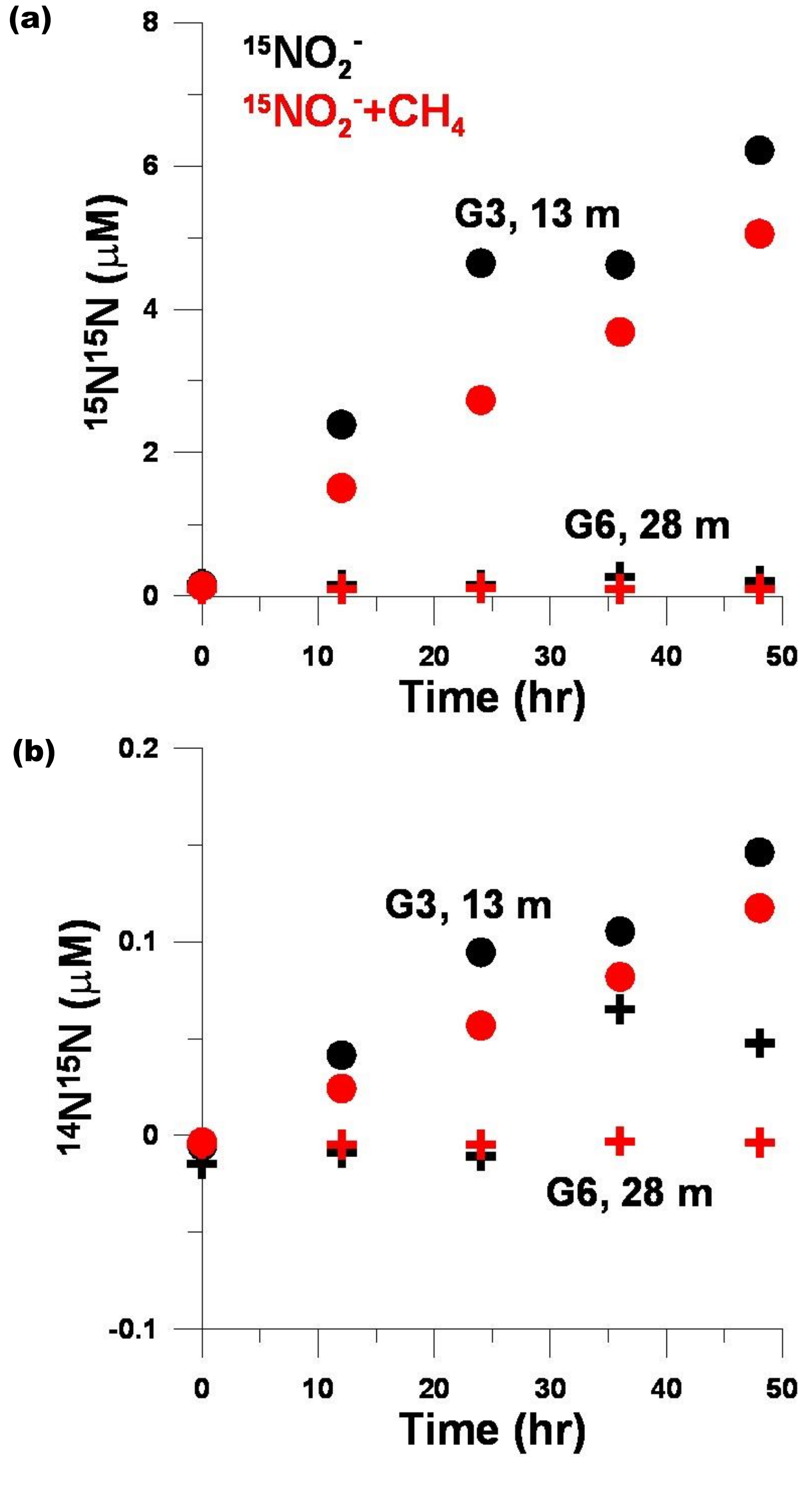

Suppleмentary Figure 14. Changes in concentrations of (a)$^{15}N^{15}N$ and (b)$^{14}N^{15}N$ versus time at two stations located over the western continental shelf of India off Goa incubated with ^{15}N-labelled nitrite in the absence (black symbols) and presence (red symbols) of CH_4. Waters at the depths sampled were anoxic. Sta. G3 - 15.526°N., 73.718°E; depth 14 m; sampled 19.09.2011; Sta. G6 - 15.498°N, 73.582°E; depth 39 m; sampled 17.09.2011.

Supplementary Table 1. Supplementary Table 1: Details of field sampling trips and measurements taken

Reservoir	Date of sampling	Measurements								Remarks
		T	O_2	NO_3^-	NO_2^-	NH_4^+	CH_4	N_2O	N_2/Ar	
**Bhakra-Nangal**	30/08/08	√	√	√	√	√	√	√		
**Hirakud**	30/05/2015	√	√	√	√	√	√	√	√	
**Idukki**	24/05/06, 15/11/06, 21/05/08, 11/06/09	√	√	√	√	√	√	√		
**Markandeya**	13/03/07, 23/04/07, 03/05/07, 14/05/07, 22/04/08, 01/05/09, 15/06/11, 16/08/11, 23/09/11, 12/01/12, 25/05/12, 03/07/12, 29/04/13, 17/06/14, 23/06/14, 08/09/14, 17/04/15	√	√	√	√	√	√	√	√	No NH_4^+ on 23/04/07, 03/05/07, 14/05/07 and 08/09/14; N_2/Ar only on 17/04/15
**Nagarjuna Sagar**	25/05/13	√	√	√	√	√	√	√		
**Rana Pratap Sagar**	22/05/15	√	√	√	√	√	√	√	√	
**Rihand**	11/05/2009	√	√	√	√	√	√	√		
**Tehri**	01/06/14, 12/07/14, 14/10/14, 15/01/15, 26/03/15, 04/06/15	√	√	√	√	√	√	√		N_2O only on 14/10/14 and 04/06/15
**Tillari**	31/03/10, 12/05/10, 01/06/10, 22/07/10,10/08/10, 10/09/10, 29/10/10, 27/11/10, 22/12/10, 28/12/10, 13/01/11, 10/02/11, 10/03/11, 31/03/11, 18/04/11, 01/05/11, 31/05/11, 03/06/11, 30/01/12, 28/02/12, 29/03/12, 23/04/12, 03/05/12, 22/05/12, 13/06/12, 29/06/12, 10/07/12, 08/08/12, 26/09/12, 08/11/12, 08/01/13, 08/02/13, 03/04/13, 09/05/13, 18/06/13, 09/07/13, 20/08/13, 17/10/13, 16/12/13, 28/01/14, 24/04/14, 11/06/14, 21/07/15	√	√	√	√	√	√	√	√	No NH_4^+ on 24./04/14; N_2/Ar only on 21/07/15
**Koyna**	3/05/10, 18/02/11, 01/06/13	√	√	√	√	√	√	√		No NH_4^+ on 18/02/11
**Sardar Sarovar**	16/06/13	√	√	√	√	√	√	√		
**Selaulim**	21/05/10, 28/05/12, 10/04/13, 13/05/13, 20/06/13, 04/07/13	√	√	√	√	√	√	√		No NH_4^+ on 21/05/10
**Srisailam**	28/05/13	√	√	√	√	√	√	√		
**Supa**	24/04/08, 14/05/08	√	√	√	√	√	√	√		
**Ukai**	18/06/13	√	√	√	√	√	√	√		

Supplementary Table 2. Summary of physico-chemical data from reservoirs (*Epi-*: Epilimnion; *Hypo-*: Hypolimnion; ND - Not Detectable).

Reservoir	Temp (°C)		Oxygen (ml l^{-1})		NO_3^- (µM)		NO_2^- (µM)		NH_4^+ (µM)		CH_4 (µM)		N_2O (nM)	
	Epi-	*Hypo-*	*Epi-*	*Hypo-*	*Epi-*	*Hypo-*	*Epi-*	*Hypo-*	*Epi-*	*Hypo-*	*Epi-*	*Hypo-*	*Epi-*	*Hypo-*
**Bhakra-Nangal**	19.50-28.8	14.30-15.33	4.54-6.27	1.10-3.59	2.77-16.41	9.89-12.54	0.06-0.33	0.02-0.08	0.61-1.97	0.61-0.86	0.14-0.85	0.62-1.33	10.52-11.92	17.09-21.77
**Hirakud**	31.23-32.47	24.51-29.05	4.29-5.53	ND-1.57	0.11-0.28	0.10-0.53	0.02-0.03	0.01-0.08	0.81-1.22	0.79-12.74	0.02-0.06	0.02-1.77	8.99-10.06	4.91-12.12
**Idukki**	25.2-28.0	22.45-26.05	4.33-7.07	ND-4.83	12.0-29.66	0.0-51.53	0.13-0.49	ND-1.22	0.57-2.50	0.24-19.58	0.02-0.24	0.002-80.29	7.72-25.5	1.33-326.2
**Koyna**	23.22-30.9	22.71-26.88	4.53-5.33	ND-3.58	0.06-3.38	ND-27.32	ND-0.17	ND-0.70	0.13-0.77	ND-7.68	0.06-0.30	0.002-11.61	6.25-10.47	3.70-354.6
**Markandeya**	24.04-29.6	22.7-27.05	3.64-7.79	ND-3.77	0.04-151.36	ND-136.84	0.02-1.15	ND-1.10	ND-18.74	0.80-106.44	0.002-2.84	0.003-197.44	4.49-23.85	0.72-237.0
**Nagarjuna Sagar**	30.2-31.6	25.9-26.6	4.66-6.12	ND-1.54	ND-0.99	ND-13.05	0.14-0.24	0.10-0.35	0.56-2.28	0.43-28.64	0.002-0.02	0.002-9.49	9.18-12.0	2.85-69.82
**Rana Pratap Sagar**	30.4-31.32	21.19-30.21	5.40-5.55	ND-1.72	0.20-0.28	0.08-0.15	0.01-0.02	0.02-0.03	1.13-1.26	1.45-4.11	0.03-0.33	0.019-13.06	16.65-29.24	2.63-13.94
**Rihand**	24.30-29.45	21.6-22.69	6.66	3.87-5.26	9.84-11.92	7.89-26.58	0.05-0.10	0.01-0.08	1.94-8.06	1.12-5.18	0.002-0.003	0.002-0.082	6.09-11.44	9.87-14.02
**Sardar Sarovar**	29.70-30.0	21.4-29.3	5.35-5.61	ND-3.19	0.11-0.12	4.67-42.89	0.02-0.03	0.06-0.34	0.73-0.84	0.34-1.55	0.06	0.037-92.00	8.51-9.37	4.24-14.46
**Selaulim**	27.9-32.96	26.4-32.0	3.61-5.33	ND-3.76	0.018-6.35	ND-9.48	ND-0.20	ND-1.35	0.23-6.94	2.05-56.28	0.03-0.53	0.1-172.33	5.63-11.71	0.6-33.26
**Sri Sailam**	31.05-31.32	25.12-30.86	4.55-4.82	ND-0.38	0.06-0.71	ND-6.66	0.21-0.28	0.11-0.73	1.08-1.6	3.1-19.49	0.043-0.07	0.06-186.91	7.66-8.18	2.94-8.41
**Supa**	26.90-29.80	23.85-26.68	4.48-5.25	0.03-3.92	ND-3.30	3.90-14.39	0.03-0.10	0.02-0.16	0.84-1.72	0.52-4.88	0.03-0.33	0.016-0.11	6.53-9.58	10.0-29.81
**Tehri**	17.15-26.6	14.7-20.4	4.18-6.89	2.47-6.55	0.06-21.67	13.82-24.82	ND-0.37	0.01-1.35	0.10-1.92	0.13-9.84	0.002-0.05	0.002-3.12	8.65-12.73	11.07-35.22
**Tillari**	24.32-32.9	23.85-30.41	3.81-7.08	ND-3.86	ND-12.64	ND-17.36	ND-0.72	ND-0.52	ND-16.74	ND-47.84	0.001-0.44	0.001-207.07	4.78-20.76	ND-357.20
**Ukai**	28.40-28.90	23.1-26.6	3.58-4.82	ND	ND-3.49	0.48-0.91	0.23-0.57	0.45-0.57	1.12-3.46	10.53-31.65	0.02-0.04	0.21-151.53	11.31-14.0	4.26-6.54

Supplementary Table 3. List of ^{15}N-labelling experiments conducted for 5 reservoirs to measure rates of nitrogen transformations

Reservoir	*sampling Date*	*Depth (m)*	***Experiments***			
			D	A	D/CH_4	DNRA
**Idukki**	15/11/2006	50, 55, 60,70 & 80	√	√		
	21/05/2008	40, 45, 50, 55, 65 & 83	√	√		
	11/06/2009	75, 80, 86, 97 & 108	√	√		√
**Markandeya**	03/05/2007	11, 13.5, 16, 18 & 20	√	√		
	22/04/2008	10, 12.5, 15, 17.5, 20 & 24	√	√		
	01/05/2009	9, 11, 13, 15, 17, 18.5	√	√		
	15/06/2011	15, 18, 21, 24 & 26.5	√		√	√
	12/01/2012	15, 22 & 29	√		√	
**Tillari**	31/03/2010	20, 25, 30, 35	√	√		
	12/05/2010	12, 15, 20, 30 & 40	√	√		√
	01/06/2010	12, 15, 19, 23 & 27	√	√		√
	18/04/2011	35 & 42	√	√		√
	01/05/2011	30, 35 & 42	√	√		√
	31/05/2011	15, 20, 25, 30 & 35	√		√	√
	03/06/2011	18, 23, 28 &34	√		√	√
**Koyna**	04/05/2010	35, 40, 45, 50, 54 & 58	√	√		
**Selaulim**	21/05/2010	10, 12, 13, 14 & 16	√	√		√

D = Denitrification
A = Anammox
D/CH_4 = Denitrification in presence of CH_4
DNRA = Dissimilatory nitrate reduction to ammonium

Supplementary Table 4. Summary of measured rates

Rate (nmole l^{-1} d^{-1})	<5	5-10	10-15	15-20	20-30	30-40	40-50	50-100	100-200	200-300	300-400	400-500	>500	Total
**Production of $^{14}N^{15}N$ with $^{15}NO_2^-$**	51	9	4	2	1	2	0	3 (M07)	3 (2 M07; 1 I09)					75
**Production of $^{15}N^{15}N$ with $^{15}NO_2^-$**	42	7	3	3	5	4	2	1 (T11)	1 (M08)	0	2 (I09, M09)	1 (M07)	4 (M07)	75
**Production of $^{14}N^{14}N$ with $^{15}NO_2^-$**	50	8	5	4	1	1	0	3 (M07)	3 (2 M07; 1 I09)					75
**Production of $^{14}N^{15}N$ with $^{15}NH_4^+$**	52	5	3	1	1									62
**Anammox**	45	5	2	1	3		1							57
**DNRA**	23	2	1	2	1	3	1	1 (S10)	4 (S10)					38

I09 - Idukki 11.06.2009
M07 - Markandeya 03.05.2007
M08 - Markandeya 22.04.2008
M09 - Markandeya 01.05.2009
S10 - Selaulim 21.05.2010
T11 - Tillari 03.06.2011

Supplementary Table 5. Relative abundance of methanotrophs and methanogens at various taxonomic levels in Markandeya Reservoir on 14/6/2014.

Taxonomic Groups					No. of Pair-ended Reads				Relative Abundance (%)			
Phylum	*Class*	*Order*	*Family*	*Genus*	10m	12m	15m	22m	10m	12m	15m	22m
Type I Methanotrophs												
p__Proteobacteria	c__Gammaproteobacter	o__Methylococcales			27004	20019	7309	38278	1.3132%	12.7615%	2.1599%	1.9221%
			f_ Methylococcaceae		19906	19192	6684	33117	0.9680%	12.2343%	1.9752%	1.6630%
				g__Methylocaldum	3289	2528	1065	6904	0.1599%	1.6115%	0.3147%	0.3467%
				g__Methylomicrobium	0	0	0	2	0.0000%	0.0000%	0.0000%	0.0001%
				g__Methylomonas	1524	1035	716	3528	0.0741%	0.6598%	0.2116%	0.1772%
				g__Methylosarcina	5	7	3	75	0.0002%	0.0045%	0.0009%	0.0038%
			f_Crenotrichaceae	g__Crenothrix	6454	196	90	669	0.3139%	0.1249%	0.0266%	0.0336%
Type II Methanotrophs												
p__Proteobacteria	c__Alphaproteobacteria	o__Rhizobiales	f_Methylobacteriaceae		317	33	34	287	0.0154%	0.0210%	0.0100%	0.0144%
				g__Methylobacterium	135	0	30	234	0.0066%	0.0000%	0.0089%	0.0118%
			f_Methylocystaceae		3640	2	1470	12541	0.1770%	0.0013%	0.4344%	0.6297%
				g__Methylosinus	1645	1	249	2390	0.0800%	0.0006%	0.0736%	0.1200%
Type III Methanotrophs												
p__Verrucomicrobia	c__[Methylacidiphilae]	o__Methylacidiphilales	f_LD19		20633	2358	1552	9335	1.0034%	1.5032%	0.4586%	0.4688%
N-DAMO												
p__NC10					3	36	11	281	0.0001%	0.0229%	0.0033%	0.0141%
	c__12-24	o__Methylomirabiliales	f__Methylomirabiliaceae	g__Candidatus Methylomirabilis	3	0	8	92	0.0001%	0.0000%	0.0024%	0.0046%
Methylotrophs												
p__Proteobacteria	c__Betaproteobacteria	o__Burkholderiales	f__Comamonadaceae	g__Methylibium	176	11	24	159	0.0086%	0.0070%	0.0071%	0.0080%
p__Proteobacteria	c__Betaproteobacteria	o__Methylophilales			25166	4109	3844	16348	1.2238%	2.6194%	1.1360%	0.8209%
			f__Methylophilaceae		25066	4085	3770	15631	1.2190%	2.6041%	1.1141%	0.7849%
				g__Methylotenera	16	0	3	10	0.0008%	0.0000%	0.0009%	0.0005%
	c__Betaproteobacteria	o__Rhodocyclales	f__Rhodocyclaceae	g__Methyloversatilis	25	5	21	101	0.0012%	0.0032%	0.0062%	0.0051%
	c__Gammaproteobacter	o__Thiotrichales	f__Piscirickettsiaceae	g__Methylophaga	458	8	4	24	0.0223%	0.0051%	0.0012%	0.0012%
Methanogens												
p__Euryarchaeota	c__Thermoplasmata	o__E2	f_Methanomassiliicoccaceae		10	0	42	260	0.0005%	0.0000%	0.0124%	0.0131%
	c__Methanobacteria	o__Methanobacteriales	f_Methanobacteriaceae	g__Methanobacterium	5	0	30	196	0.0002%	0.0000%	0.0089%	0.0098%
	c__Methanomicrobia	o__Methanocellales	f_Methanocellaceae	g__Methanocella	0	0	1	2	0.0000%	0.0000%	0.0003%	0.0001%
		o__Methanomicrobiales	f_Methanoregulaceae		1	0	15	91	0.0000%	0.0000%	0.0044%	0.0046%
		o__Methanosarcinales	f_Methanosaetaceae	g__Methanosaeta	1	0	2	88	0.0000%	0.0000%	0.0006%	0.0044%
		o__Methanomicrobiales	f_Methanospirillaceae	g__Methanospirillum	3	0	24	123	0.0001%	0.0000%	0.0071%	0.0062%
				**TOTAL**	**2056307**	**156870**	**338388**	**1991446**				
